# Diagnostic and Therapeutic Strategies for Brain Metastases from Unknown Primary Tumors: A Comprehensive Review

**DOI:** 10.3390/jcm15072642

**Published:** 2026-03-31

**Authors:** Dan M. Visarion, Ioana Petrescu, Viorel M. Pruna, Radu M. Gorgan

**Affiliations:** 1 Department of Neurosurgery, “Carol Davila” University of Medicine and Pharmacy, 020021 Bucharest, Romania; marius-dan.visarion@drd.umfcd.ro (D.M.V.);; 2 Department of Neurosurgery, “Bagdasar-Arseni” Clinical Emergency Hospital, 041915 Bucharest, Romania; mironioana@gmail.com

**Keywords:** brain metastasis, cancer of unknown primary, immunohistochemistry, treatment of brain metastases, molecular markers of CUP, BM-CUP

## Abstract

Brain metastases represent a heterogenous group of tumors, with distinct characteristics and prognosis, depending on the primary malignancy and on the patient performance status. In up to 28% of cases, neurological symptoms from a brain metastasis are the initial clinical manifestation of the primary cancer. If the primary neoplasm remains unidentified, patients are diagnosed with Brain Metastases for Cancer of Unknown Primary (BM-CUP). While brain metastases are far more common than primary brain tumors, CUP represents a rare malignancy, accounting for 2–5% of all cancer cases. It is characterized by an aggressive clinical course, rapid metastatic spread, low response rate to chemotherapy, and poor prognosis. Understanding BM-CUP remains a challenge, and being such a rare disease, there is still a significant lack of consensus regarding the management of these patients. Nevertheless, recent progression in diagnostic techniques, immunohistochemistry, and genomic sequencing open a new horizon to personalized therapies in selected cases. However, limitations persist, including biological heterogeneity and limited access to genomic sequencing, often leading to the use of empirical treatment. This review synthesizes recent advancements in the diagnosis and management of BM-CUP, highlighting the importance of a structured therapeutic approach for optimizing survival outcomes and preserving quality of life in this challenging patient population.

## 1. Introduction

Brain metastases are the most common brain tumors, surpassing the incidence of primary brain tumors, with a current ratio of up to 5:1 and an increasing trend [1]. Up to 20% of oncological patients develop symptomatic brain metastases, while autopsy series have identified them in up to 50% of cases, although the majority remain asymptomatic [1,2]. A significant part of these patients, up to 28%, present with symptoms caused by brain metastases as their first clinical manifestation [3,4]. The most prevalent primary sources of brain metastases are: lung (40–60%), breast cancer (10–40%), melanoma (10–30%), renal cancer (5–10%), cancer of unknown primary (CUP) (5–15%), colorectal and digestive cancer (3–6%), and other neoplasms (2–10%) [1,3,5]. CUP is a heterogeneous category defined by the clinical detection of metastases while the primary tumor remains undiagnosed by current imaging investigations [1,2].

Patients presenting with brain metastases as their initial clinical symptom can be categorized into two distinct groups: those where imaging reveals the primary site, and those where systemic malignancy remains undetected. The latter are typically hospitalized with the suspicion of primary brain tumor [1]. Cases with histologically confirmed brain metastases where the primary tumor remains unidentified following a standardized diagnostic work-up are categorized as brain metastases from unknown primary tumors (BM-CUP) [6].

The median survival for these patients is 11 months from the time of diagnosis. The prognosis is significantly poorer for those with brain metastases arising from CUP (BM-CUP) than for those developing brain metastases during the progression of a previously diagnosed neoplasia, as these cases often involve malignancies with aggressive evolution and a suboptimal response to chemotherapy [5]. In up to 15% of cases involving brain metastases, the primary malignancy remains occult despite diagnostic efforts [1,2].

The present article synthesizes recent advancements in the diagnosis and management of BM-CUP, a field that continues to evolve alongside advancements in molecular and genetic diagnostic techniques. Nevertheless, this particular category of brain tumors remains a significant challenge in neuro-oncology. Neurosurgical treatment plays a pivotal role in the management of BM-CUP, not only for achieving local control, but also because accurate tissue sampling is the essential first step toward personalized therapies. Nonetheless, limited accessibility and difficulties in data interpretation often delay the initiation of targeted therapies, contributing to a poor prognosis for these patients [4,6].

## 2. Materials and Methods

**Figure 1:**
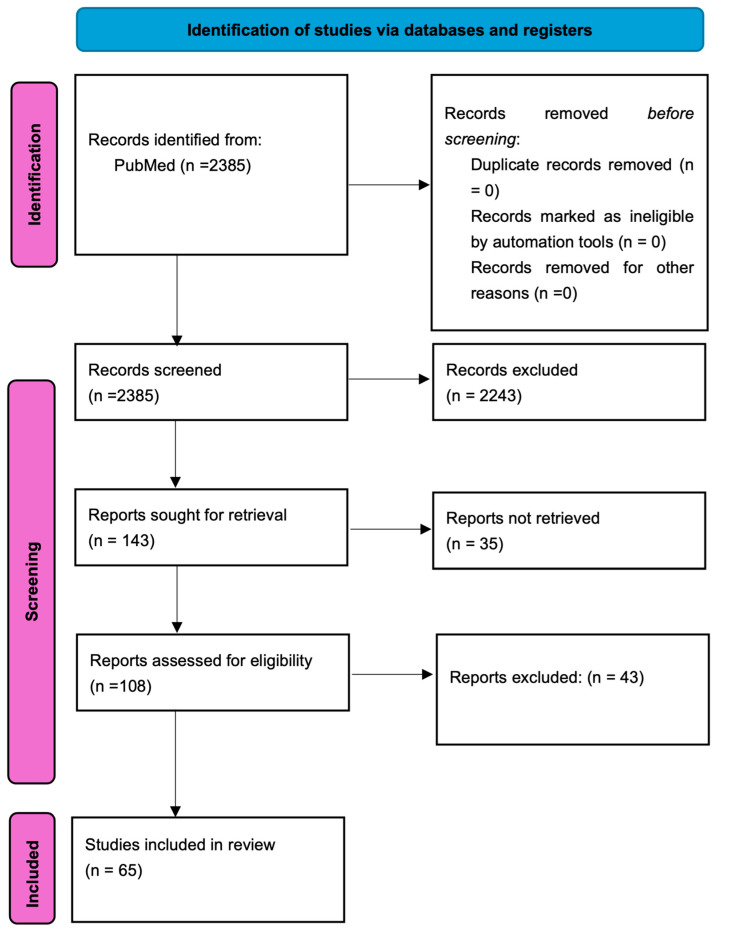
Flowchart depicting the selection of the studies included in the present review.

The authors performed extensive literature research using PubMed on articles published up to 1 January 2026. The search string on PubMed was: “brain metastasis” OR “brain metastases” AND “cancer” OR “neoplasm” AND “unknown”. In further searches, the following terms were added “unknown primary tumor” OR “unknown primary cancer” AND “target therapy”, “management”, “treatments” and “clinical trials”. Only articles written entirely in English (full-text articles) were included. A total of 2385 articles were identified and their titles and abstracts were screened; of these, 243 articles were selected as representative for the review’s topic. The inclusion criteria for the selected articles were the incidence and clinical characteristics of CUP and especially in the BM-CUP subgroup, as well as the diagnostic standardization for patients with BM-CUP. Furthermore, we assessed the prognosis of these patients compared to other brain metastases and other CUP patients. Another key topic was the identification of immunohistochemical and genetic markers in these lesions, highlighting their potential in order to facilitate diagnosis and enable targeted therapy. The exclusion criteria consisted of outdated articles that no longer provide relevant information, redundant studies and those which did not include BM-CUP patients in their analysis. The reference lists of all the selected articles were screened to detect additional studies from the same area of interest. Therefore, after screening the titles and abstract, 65 studies were deemed relevant to our research topic and included in this review. The search process is summarized in Figure 1.

## 3. Characteristics of Cancer of Unknown Primary (CUP)

The entity known as CUP has been subject to various nomenclatures and definitions, resulting in a heterogeneous group of scientific medical articles that is challenging to categorize. The most widely accepted definition remains the clinical detection of metastatic disease in the absence of a diagnosed primary tumor following a comprehensive imaging diagnosis [6,7,8,9,10]. The widely accepted time interval is up to 3 months during which the systemic cancer cannot be identified through imaging [11,12]. The development of diagnostic methods, histopathological and surgical techniques has led to a more precise and accurate identification of the primary lesion, resulting in a decreased incidence of these cases; however, 2–5% of cases still fall under the category of CUP syndrome. There are reported cases in which the primary lesion could not be identified even upon performing an autopsy [13]. The patients with brain metastases from CUP account for up to 10% of all brain metastases cases. Surgical intervention aiming resection or biopsy remains the main method that can confirm this diagnosis and plays a key role in patient management [6]. The European Society for Medical Oncology (ESMO) has released a guideline for the correct evaluation and diagnosis of CUP (Table 1) [14]. This encompasses comprehensive clinical examination, which must include head, neck, thoracic, abdominal, breast, and pelvic/gynecological examinations, aimed to identify clinical signs of a primary tumor [14,15,16]. Laboratory tests include both standard and patient-specific examinations. Common tests include a full blood count, liver and kidney function tests, electrolytes and LDH levels. Specific examinations include tumor markers, which have limited diagnostic value and are indicated only when symptoms suggest a specific neoplasm, such as: β-hCG and alpha-fetoprotein (α-FP) for liver involvement (hepatocellular carcinoma) or midline lymph node involvement (germ cell tumors), PSA for suspected prostate cancer, calcitonin for medullary thyroid carcinoma and catecholamines for neuroendocrine tumors [16,17].

Imaging evaluation is the primary modality for localizing a potential primary tumor. Contrast-enhanced CT is the investigation of choice for detecting a primary lesion in the chest, abdomen, and pelvis, or other metastases within these segments. MRI is the gold standard investigation required for the diagnosis of brain metastases, offering the highest sensitivity in their identification. Mammography or breast ultrasound are essential for the evaluation and detection of breast cancer. Should initial examinations remain negative, a whole-body FDG-PET may be considered, depending on availability. While FDG-PET has not proven superior to standard CT for primary tumor localization, it enhances the detection of additional systemic metastases, thereby refining staging and informing oncological management. Standard histological evaluation, combined with immunohistochemistry (IHC) and Next-Generation Sequencing (NGS), represent a critical diagnostic framework for CUP, enabling the implementation of personalized molecular therapies [6,18,19,20].

## 4. Biological Models of Cancer of Unknown Primary

Understanding the origin and development of CUP has always been a subject of interest, yet it has not been fully explained. At present, the most widely accepted theories are the following [21]:

- Parallel progression model: this theory proposes that early-stage tumor cells disseminate into the circulation shortly after their emergence in the primary organ. The occult nature of the primary site is hypothesized to result from immune-mediated clearance or cellular dormancy. Notably, the metastatic clones undergo significant phenotypic alterations, enabling them to bypass host defense mechanisms.
- Unknown primary metastatic disease model: the foundation of this theory is based on the supposition that a primary neoplasm does not exist; instead, the tumor cells originate from pluripotent stem cells that have undergone a major phenotypic transformation. These cells lack the specific characteristics of the tissue in which they are found, a theory supported by the cellular alterations observed in the metastases.

The most frequent molecular and genetic alterations associated with and implicated in CUP include chromosomal alterations, resistance to apoptosis, insensitivity to growth-inhibitory signals, sustained angiogenesis, epithelial–mesenchymal transition (EMT), and evasion of immune attack. While these changes are also involved in the development of primary neoplasms, in the case of CUP, these molecular and genetic alterations are likely so significant that they may no longer exhibit the specific characteristics associated with the primary tumors [6,21].

## 5. IHC Profiling for Primary Tumor Identification

The histological and immunohistochemical evaluation of tumor fragments is fundamental in identifying the primary lesion when it cannot be detected through conventional imaging techniques. The EANO guidelines support and emphasize the importance of this analysis, as it aids in characterizing the primary lesion—a crucial step for the initiation and adjustment of oncological treatment. Despite the use of extensive immunohistochemistry combined with new molecular techniques, in up to 11% of cases, the histological nature of the primary cancer still cannot be identified [22,23]. Adenocarcinoma is the most frequent histological type of cancer associated with brain metastases from cancer of unknown primary (BM-CUP), followed by squamous cell carcinoma [16]. From a diagnostic perspective, cytokeratin markers CK7 and CK20 play a crucial role in the staging of carcinomas. Initial immunohistochemical evaluation begins with a baseline panel: CEA for lung and gastrointestinal cancer, TTF-1 for lung adenocarcinoma, CA 19-9 for gastrointestinal cancer, CK20 for colon cancer, CA125 for ovarian cancer, BCA 225 for breast cancer, PAX8 for gynecological, renal and thyroid cancers, PSA for prostate cancer, and HMB-45 for melanoma. In summary, the immunohistochemical testing of patients with brain metastases originating from CUP should include: CK AE1/AE3, CK7, CK18, CK20, vimentin, S100 protein, TTF-1, CA15-3, CA125, CA19-9, and PSA (for males) [16]. The limitations of IHC are often due to small, poorly differentiated histological samples disseminated within normal tissue; consequently, significant differences may exist between histological areas, especially when compared to other metastases or the primary lesion. An extensive immunohistochemical examination is structured in Table 2.

## 6. The Evolution of Molecular and Genomic Diagnostics

Molecular and genomic diagnosis represents a new important part in the diagnosis and treatment of patients with CUP, aiming to increase the detection rate of primary lesions and to identify potential targeted therapies. NGS techniques and gene expression platforms (such as EPICUP and CancerTYPE ID) collectively enable the genetic and epigenetic analysis of the tumor, providing insights into its origin and biological behavior. Gene expression platforms are assays that identify specific genes to facilitate the diagnosis of the primary lesion, with an accuracy range of 75% to 92%, as seen in the CancerTYPE ID analysis. EPICUP is an analysis based on DNA methylation patterns, demonstrating an accuracy of up to 95% in selected cases through the analysis of tumor DNA alterations [15,19,25].

The most frequent genes identified in patients with CUP have been reported as TP53, RAS, MYC, CDKN2A, and BRAF; however, these are not tissue-specific, which limits the utility of Gene Expression Profiling (GEP) in localizing the primary tumor [19]. The identification of key genomic alterations, such as BRCA1 and BRCA2, enables specific therapy with PARP inhibitors. Similarly, the NTRK gene offers the possibility of targeted therapy with entrectinib or larotrectinib, while detecting other genomic changes like BRAF, EGFR, IDH1/2, or HER2 opens avenues for biological therapies. Beyond genomic alterations that provide targeted options, genes like STK11 or KEAP1 can be identified, which are associated with primary resistance to biological therapy [16,17,19,21]. In patients with BM-CUP, the applications of these techniques are still limited, due to high costs and limited global accessibility. Another limitation is represented by small tissue samples typically obtained from biopsies; therefore, a definitive diagnosis is often challenging. Furthermore, these patients frequently present with a poor performance status, requiring oncological intervention, which often leads to the use of conventional chemotherapy. In cases of CUP with systemic metastases, multiple small-scale studies have demonstrated the applicability and utility of these techniques, identifying the primary histological type in up to 80–90% of cases [16,19].

The advances in molecular and genomic diagnostics serve as a cornerstone for modern oncological management in BM-CUP cases. Expanding these analyses is vital for establishing targeted protocols that can significantly improve patient outcomes and prognosis.

## 7. Therapeutic Strategies for CUP and BM-CUP Patients

### 7.1. Management of Patients with CUP

The management of patients with CUP consists of obtaining a tissue fragment (biopsy or tumor resection) for histological confirmation, followed by systemic pharmacotherapy and radiotherapy [5,14]. The clinical course of patients diagnosed with systemic metastases from CUP follows two patterns: the first involves an unfavorable prognosis, where clinical findings and immunohistochemical results cannot identify the primary lesion; the second pattern shows a favorable prognosis, where the primary lesion may be suspected [6,16]. This latter group exhibits a positive response to conventional oncologic treatment and follows a clinical evolution similar to patients with a confirmed primary lesion. In most frequent cases—approximately three-quarters of all instances—the clinical course is unsatisfactory and is associated with several characteristics: age > 65 years, male gender, KPS < 70, multiple comorbidities, multiple metastases at the time of diagnosis (specifically brain, bone, or pleural involvement), and the adenocarcinoma histological subtype. For patients diagnosed with CUP, conventional chemotherapy used to be the primary mainstay, but with modest results; however, targeted therapy guided by molecular profiling might offer hope for these patients [6,14,16].

### 7.2. Management of Patients with BM-CUP

**Figure 2:**
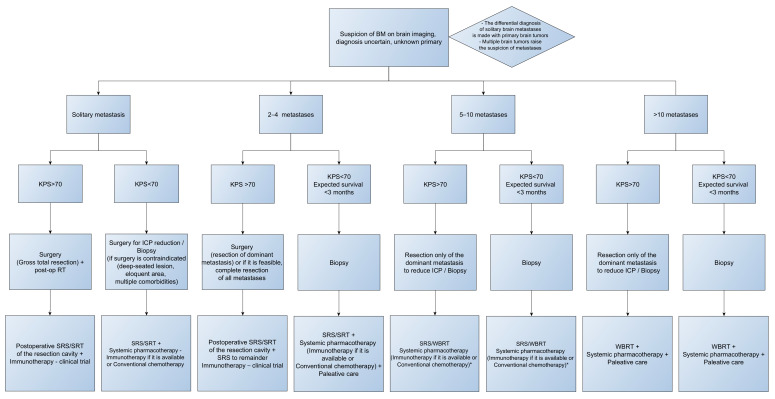
Therapeutic algorithm for the management of BM-CUP. * The combination of WBRT and Immunotherapy necessitates rigorous surveillance.

In patients with BM-CUP, surgical intervention is of particular importance in their management; it provides the tumor tissue necessary for diagnostic confirmation, reduces intracranial pressure, and, in cases of single or multiple brain metastases where complete resection is achieved, it ensures local tumor control [22]. The management of patients with BM-CUP is based on retrospective studies with small sample sizes; consequently, data regarding this patient group remain limited, reporting an overall survival expectancy of up to 9 months [11,27]. Future phase II/III prospective studies are expected to demonstrate the efficacy of current systemic therapies, potentially refining the therapeutic management of these patients. When comparing survival of patients with BM-CUP to patients with BM from known primary systemic cancer, not all studies reported a statistically significant difference [6,11]. Overall, patients with BM-CUP who present with extracerebral metastases, older than 65, with deep-seated lesions, KPS < 70 or harboring multiple brain metastases have a guarded prognosis. KPS score is a very important factor in the therapeutic algorithm of patients with brain metastases. Patients presenting with poor clinical status (KPS < 70) are often candidates for biopsy, except for those harboring posterior cranial fossa tumors, who benefit from surgery regardless of the KPS score. Conventional chemotherapy for BM-CUP shows a 2-year survival rate of 12% [28]; the high mortality rate in this group is primarily associated with the extension of systemic disease rather than intracranial progression [18]. Local treatment, consisting of surgery and radiotherapy, is tailored based on specific factors such as the number of intracranial lesions, tumor volume, location of the tumor (deep, eloquent areas versus cortical ones), and the patient’s performance status. In patients with brain metastases that are not candidate for surgery, stereotactic radiosurgery is reported to obtain the same local control as surgical resection [29,30,31]. According to data reported in the literature, Whole-Brain Radiotherapy (WBRT) has played a significant role in the management of patients with BM-CUP; furthermore, recent studies have analyzed its use in combination with surgical treatment, which has offered patients a favorable clinical outcome [32]. Figure 2 illustrates a detailed therapeutic algorithm for patients with BM-CUP, based on the two most critical decisional factors: the number of metastases and the patient’s KPS score, to guide personalized clinical management. The tree chart from Figure 2 was generated using yED Graph Editor 3.25.1. 

The extent of resection (EOR) and the surgical technique have been evaluated as prognostic factors in numerous studies; thus, most research has associated gross total resection (GTR) and en-bloc resection with superior survival rates compared to subtotal resection (STR) and to piecemeal technique [6,11,33,34].

## 8. New Perspectives in Current Practice

For patients with CUP, the main objective is to obtain detailed molecular profiling and immunohistochemical analyses to provide opportunities for targeted biological therapies. The efficacy of these techniques and the introduction of personalized treatment were initially demonstrated in published case reports [6,15,21], which supported and proved the effectiveness of these therapies and their superiority over conventional chemotherapy. Binder et al. reported that relevant molecular profiles were identified in 85% of CUP patients, and approximately 50% of these patients could potentially receive targeted therapy [35].

### New Perspectives in BM-CUP Target Therapies

Patients with BM-CUP were initially excluded from these clinical trials due to their associated unfavorable prognosis; consequently, data related to this specific cohort remain poorly represented. The main targeted therapies that can be utilized for these patients are summarized in Table 3.

The importance of identifying these genomic alterations through NGS has led to the development of therapies like as entrectinib and larotrectinib. These agents block autophosphorylation and downstream intracellular signaling, demonstrating their efficacy by improving survival rates with minimal or well-tolerated adverse reactions [35,40,41]. Furthermore, PARP inhibitor therapy, which targets BRCA1/2 gene mutations at the DNA level, offers an improved prognosis for these patients when combined with conventional chemotherapy.

Immunotherapy also plays a crucial role in their treatment, particularly immune checkpoint inhibitors (ICIs), which have demonstrated the ability to cross the blood–brain barrier and have shown intracranial efficacy in numerous studies [42,43]. The efficacy of pembrolizumab in combination with platinum-based chemotherapy was demonstrated in the KEYNOTE-189 clinical trial (NCT02578680) in patients with NSCLC, achieving an overall survival of 19.2 months versus 7.5 months in patients with brain metastases [44]. Similar results were obtained in other clinical trials and were further supported by up to 5-year follow-up of patients in the KEYNOTE-189 study [44,45]. A meta-analysis comparing immunotherapy combined with chemotherapy or radiotherapy versus standard therapy or immunotherapy alone demonstrated that combination therapy provides an improved progression-free survival (PFS) and an overall survival (OS). Another important finding of the meta-analysis is that there is no statistically significant difference between the types of ICI therapies: PD-L1, PD-1, and CTLA-4 [46]. The phase II Astro-Brain trial, which evaluates the therapeutic response to astrolizumab, a PD-L1 inhibitor antibody, in combination with carboplatin and pemetrexed in patients with non-squamous NSCLC and inoperable brain metastases, showed a favorable response, with a PFS of 6.9 months for intracranial progression and an OS of 11.8 months [47]. However, it should be emphasized that PD-L1 expression is a favorable prognostic factor in patients with brain metastases treated with ICIs [43]. Another phase II clinical trial (NCT02886585), which evaluated the intracranial efficacy of pembrolizumab in patients with brain metastases regardless of the primary tumor origin, including CUP, demonstrated a benefit for these patients, thereby providing an opportunity for the development of further studies aimed at expanding the indications of these therapies [48]. All these studies highlight the importance of these therapies in patients with brain metastases and suggest that they are likely to play a significant role in the management of patients with BM-CUP, with the potential to improve their prognosis.

## 9. Conclusions

The therapeutic management of patients with BM-CUP has undergone multiple changes and continues to present updates and gaps regarding diagnostic methods for the primary lesion. Molecular techniques have evolved and been rapidly implemented for these patients, leading to their referral toward molecular targeted therapies. The main objectives remain improving both overall survival and quality of life for these patients.

## Figures and Tables

**Table 1:** Evaluation and Diagnosis Algorithm for BM-CUP [14,15].

Category	Investigation/Procedure	Specific Details & Indications
Clinical Assessment	Medical History	Includes medical and surgical history, family history, toxic habits (smoking/alcohol), and occupational history.
	Physical Examination	Complete organ system review: peripheral lymph nodes, breast, skin, and pelvic-genital examination.
Laboratory tests	Blood Panels	Complete blood count (CBC) and biochemistry (including calcium and LDH).
Imaging modalities	CT scan	Contrast-enhanced CT scan of the chest, abdomen, and pelvis.
	Breast Screening	Mammography or breast ultrasound.
	Breast MRI	Indicated if mammography is inconclusive.
	PET-CT	Whole-body 18F-FDG PET-CT for systemic staging.
	Targeted Ultrasounds	Thyroid ultrasound Testicular ultrasound (based on clinical suspicion).
Endoscopy	Bronchoscopy	Performed in case of pulmonary symptoms.
	Gastrointestinal Workup	Colonoscopy and gastroscopy if gastrointestinal symptoms occur or if CT is inconclusive.
Pathology	Biopsy	Histological confirmation of metastatic cancer.
	Histopathological Review	Comprehensive review with a specific immunohistochemistry (IHC) study.
Tumor Markers	Serum PSA	Mandatory concentration test for all men.
	Serum CA 125	Specifically for suspected gynecological/ovarian origin.
	Germ Cell Markers	Serum ά-FP and β-hCG for undifferentiated tumors in patients under 50 years old.
Other	Supplemental Tests	Targeted tests performed only in the presence of specific signs or symptoms.

**Table 2:** Immunohistochemical examination for different neoplastic diseases [16,22,23,24,25,26].

Primary Tumor Site	Specific Immunohistochemical (IHC) Markers
Lung Adenocarcinoma	CEA, EMA, CK7, TTF-1, CAM 5.2, Napsin A, Surfactant A & B
Lung (non-adenocarcinoma)	CK7, TTF-1, p63, CK5/6, Napsin A
Small Cell Lung Carcinoma	Synaptophysin, EMA, TTF-1, CD56, CK7, Ki-67
Breast Adenocarcinoma	EMA, GATA3, Estrogen Receptor (ER), CA 15-3, CEA, CK5, CK7, CAM 5.2, Mammaglobin
Endometrium	Vimentin, ER, PAX8
Germ Cell Tumors	α-FP, β-HCG, CD30, OCT3/4
Colorectal Adenocarcinoma	CEA, EMA, CA 19-9, CAM 5.2, CK20, SATB2
Stomach Adenocarcinoma	CAM 5.2, CK7, CK20, CDX2, EMA, CEA, CA 19-9
Renal Cell Carcinoma	RCC antigen, EMA, PAX8, Vimentin, CD10
Melanoma	Vimentin, Melan A, S100, HMB-45
Pancreas/Bile Duct	CDX2, CK7, CA 19-9

**Table 3:** Target therapies utilized in the treatment of BM-CUP [16,36,37,38,39,40,41].

Genomic Alteration	Targeted Treatment
ALK	Lorlatinib, Ceritinib, Alectinib, Crizotinib
RET	Pralsetinib, Selpercatinib
ROS1	Crizotinib, Lorlatinib
BRCA 1/2	Niraparib, Rucaparib
EGFR	Erlotinib, Afatinib, Dacomitinib, Gefitinib
BRAF	Encorafenib, Dabrafenib
IDH 1/2	Vorasidenib
NTRK	Entrectinib, Larotrectinib
ALK	Lorlatinib, Ceritinib, Alectinib, Crizotinib
RET	Pralsetinib, Selpercatinib
ROS1	Crizotinib, Lorlatinib
BRCA 1/2	Niraparib, Rucaparib
EGFR	Erlotinib, Afatinib, Dacomitinib, Gefitinib
BRAF	Encorafenib, Dabrafenib
IDH 1/2	Vorasidenib
NTRK	Entrectinib, Larotrectinib
ALK	Lorlatinib, Ceritinib, Alectinib, Crizotinib
RET	Pralsetinib, Selpercatinib
ROS1	Crizotinib, Lorlatinib
BRCA 1/2	Niraparib, Rucaparib
EGFR	Erlotinib, Afatinib, Dacomitinib, Gefitinib
BRAF	Encorafenib, Dabrafenib

## Data Availability

No new data were created or analyzed in this study.

## Abbreviations

The following abbreviations are used in this manuscript:

CUP	cancer of unknown primary
BM-CUP	brain metastases from cancer of unknown primary
EOR	Extend of resection
GTR	Gross total resection
ESMO	The European Society for Medical Oncology
NGS	Next-Generation Sequencing
IHC	Immunohistochemistry
EMT	epithelial–mesenchymal transition
WBRT	Whole-Brain Radiotherapy
EANO	European Association of Neuro-Oncology
LDH	Lactate-Dehydrogenase
α-FP	Alfa-fetoprotein
β-hCG	Beta-human chorionic gonadotropin
PSA	Prostate-specific antigen
FDG-PET	Fluorodeoxyglucose-Positron Emission Tomography
CK7, CK20 etc.	cytokeratin markers
CEA	Carcino-embryonic agent
GEP	Gene Expression Profiling
TTF-1	Thyroid transcription factor 1
ER	Estrogen Receptor
PFS	Progression free survival
OS	Overall survival
ICIs	immune checkpoint inhibitors
NSCLC	Non-small cell lung carcinoma
PD-L1	Programmed Death-ligand 1
PD-1	Programmed cell Death Protein-1
CTLA4	Cytotoxic T-lymphocyte-associated protein 4
